# Contact After Emergency Department Discharge by a Telehealth Transition of Care Program Is Associated With Reduced Emergency Department Revisit Rate

**DOI:** 10.7759/cureus.100433

**Published:** 2025-12-30

**Authors:** Michael B Light, Michelle Elsener, Cheng Feng, Kerri Elsabrout, Farrukh N Jafri

**Affiliations:** 1 Department of Emergency Medicine, Rutgers University New Jersey Medical School, Newark, USA; 2 WPH Cares Transitional Care, White Plains Hospital, White Plains, USA; 3 Data Analytics, White Plains Hospital, White Plains, USA; 4 Patient Care Services, White Plains Hospital, White Plains, USA; 5 Emergency Medicine, White Plains Hospital, White Plains, USA

**Keywords:** emergency department readmission, emergency medicine, readmission, telehealth, transition of care

## Abstract

Objective

The effectiveness of transition of care programs for patients discharged from the emergency department (ED) varies. This study aimed to evaluate the impact of the White Plains Hospital Cares (WPH Cares) program on repeat ED visits within 7 and 30 days after discharge. Secondary objectives included assessing patient characteristics affecting return rates, the optimal timing for telehealth outreach, and the best time to contact patients after ED discharge.

Methods

This cohort study analyzed data from patients discharged from the ED to home between May 2021 and April 2023 at a community hospital. Data analysis was conducted from March 2023 to July 2024. All discharged patients were eligible for contact by the WPH Cares program, which involves a team of nurses and social workers aiming to reach patients within 48 hours. The WPH Cares structured assessment provided follow-up support, including appointment navigation, reviewing discharge instructions, facilitating medication access, home services, and delivering disease-specific care. The primary outcome was ED return visits at 7 and 30 days.

Results

During the study period, 106,888 patients were discharged home from the ED. The WPH Cares team attempted to contact 93,933 (87.9%) of these patients, successfully reaching 52,210 (48.8%). Patients successfully contacted by WPH Cares had a lower likelihood of returning to the ED within 7 days (4.1% vs. 6.4%; OR 0.69, p<0.0001) and within 30 days (10.0% vs. 12.8%; OR 0.82, p<0.0001) compared to those not contacted.

Conclusions

Among ED patients at a single community hospital, successful contact by a multidisciplinary telehealth transitional care program was associated with lower 7- and 30-day revisit rates. Optimal outreach timing was >72 hours post-discharge and during weekday late afternoon/evening hours. These observational findings require confirmation with balanced comparison groups and prospective designs.

## Introduction

Discharging patients from the Emergency Department (ED) is an important yet challenging transition of care, requiring effective communication of diagnosis, follow-up care, and indications to return to the hospital, while providing the opportunity to address additional questions or concerns [1]. The often fast-paced nature of ED care presents several barriers to effective discharge, including distractions and time limitations [2]. Communication at discharge is further degraded by the high prevalence of limited patient and family health literacy and limited English proficiency [3, 4], inadequate delivery of key information, and discharge instructions written at inappropriately advanced reading levels [5-8]. One goal of successful transitions of care programs is to mitigate these barriers and decrease preventable revisits to the ED through safe and effective navigation post-discharge. ED visits are costly, often inconvenient for patients, and may be inappropriate if care can be provided in alternative settings, such as in ambulatory settings or telehealth [9].

Complicating effective transitions of care design is limited high-quality evidence regarding the effectiveness of ED return visit reduction programs [10]. Telehealth or telephone-based follow-up has emerged as a potential method of reducing ED return visits. However, recent studies have found that telephone follow-up with a nurse or medical student alone is unable to reduce ED revisits [11-15]. Callback programs from a clinician or enrollment into a care pathway have had more success in reducing ED visits [16,17]. Given the suggestion in the literature that more intensive, structured strategies beyond nursing callbacks may be necessary help reduce readmission or ED visit rates [18], we sought to evaluate our unique telehealth-based transitional care model, coined White Plains Hospital Cares (WPH Cares).

White Plains Hospital developed WPH Cares to improve access to care and services for patients discharged from the hospital and the ED. This program leverages a multidisciplinary team including advanced practice providers (APP), i.e., nurse practitioners and physician assistants, registered nurses (RN), social workers (SW), and care navigators (CN), overseen by a Medical Director. The model is equipped to advocate for access to medical care and services for patients, intervene clinically when indicated, and provide education, guidance, and support during the transitional period.

This manuscript outlines the evolution and impact of the WPH Cares transitional care model on patients discharged from the ED at a single site. The primary objective was to assess the association between successful patient contact by WPH Cares and repeat ED utilization at both 7 and 30 days after the initial ED visit. Our hypothesis was that successful contact by WPH Cares would be associated with lower 7-day and 30-day ED return rates. Our secondary objectives were to understand: 1) characteristics of patients contacted by WPH Cares and their association with the likelihood of return visits to the ED, 2) the ideal timing of a telehealth outreach after ED discharge and its impact on ED return, and 3) the optimal time and day of the week to attempt to contact patients post-discharge through telehealth.

## Materials and methods

Context

White Plains Hospital (WPH) is a 292-bed not-for-profit community hospital and member of the Montefiore Health System, located in White Plains, New York, and has a 21% admission rate. The hospital has been Magnet® designated since 2012 and achieved 5-star recognition by the Centers for Medicare & Medicaid Services in 2022 and 2023. In 2022, approximately 52,000 patients met criteria, i.e., were discharged home or with home care from the ED, with 59,000 meeting criteria in 2023, and a projected volume of 61,000 for 2024.

WPH Cares is a hospital-based multidisciplinary telehealth-based transitional care program that was initiated in 2018 and has evolved to focus on four strategic aims: 1) provide data-driven, innovative solutions to ensure that patients have access to services when they need them, 2) leverage hospital and community resources to decrease both 30-day hospital readmissions as well as preventable ED utilization, 3) improve access for patients to medical care and services while elevating the patient experience and 4) empower patients to be proactive about monitoring and managing their health. This program provides transitional care after both inpatient and ED visits. The interventions for the WPH Cares inpatient program have demonstrated decreases in total and disease-specific readmissions, and its workflows are outlined in Elsener et al, 2023 [19]. The interventions listed below are specific to ED patients.

Intervention

Every patient discharged from the ED to home starting in January 2018 was a candidate for participation in the WPH Cares program. Exclusion criteria were patients discharged to rehabilitation facilities, nursing homes, group homes, or assisted living facilities. WPH implemented a new electronic health record (EHR) in May 2021. Due to difficulty accessing patient data prior to this time, demographic data and analysis are provided only from May 2021 onwards.

For ED patients, there are three main facets to the WPH Cares structured outreach: 1). Clinical Outreach: A team consisting of RNs and SWs attempts to telephonically contact all patients discharged home from the ED, with the goal of contact within 48 hours of ED discharge. For those unable to be contacted, electronic portal messages are sent if patients have an activated account through the hospital's EHR. Once contacted, structured assessments focus on 1) follow-up appointments, 2) review of discharge instructions, 3) access to medications, 4) assessment of home services and social needs, and 5) predefined questions based on discharge diagnosis (e.g., new onset atrial fibrillation, COVID-19 diagnosis, etc.). RNs prioritize clinical cases while SWs focus on social determinants of health needs and home services. 2). Access Navigation: A non-clinical team consisting of patient access associates and referral navigators connects patients with provider offices and secures timely appointments based on the requests from the ED team. Referrals to this team are made either directly from the ED staff or from the Clinical Outreach team when identifying a patient in need of assistance with follow-up appointments. 3). Clinical Escalation: The Medical Director and APPs manage clinical escalations from the Clinical Outreach team or requests for provider telehealth outreach from ED providers. Services that support this program include coordination with the hospital’s outpatient Infusion Center for the administration of intravenous fluids and antibiotics, community paramedicine programs for home visits, and leveraging hospital outpatient consultants for telehealth visits.

Collaboration with community stakeholders

The above team members also leverage relationships built with key healthcare stakeholders to review and develop care plans for at-risk patients. The group consists of the visiting nurse service, local cancer centers, ambulatory care managers, and our family health center. This team helps to coordinate medication access, home services, transportation for appointments, access to charity care, palliative medicine referrals, and mental health services. Relationships were initially developed on an ad hoc basis to address specific patient needs identified through the WPH Cares program. Over time, these collaborations became formalized through shared workflows, defined referral processes, and regular communication channels between hospital leadership and community partners.

Development of intervention

The WPH Cares transitional care workflows were developed internally by a multidisciplinary task force comprising nursing, care management, social work, pharmacy, and medical leadership. The framework was informed by evidence-based transitional care models, including the University of Pennsylvania’s Transitional Care Model, Boston University’s Project RED (Re-Engineered Discharge), and the Society of Hospital Medicine’s Project BOOST [20-22]. These programs provided the foundation for our approach, which was subsequently adapted to the emergency department (ED) population and refined through internal quality improvement cycles.

Key changes in workflow from May 2021 to April 2023

Starting in January 2023, the ED volume surpassed our RNs’ and SWs’ capacity to outreach 100% of patients discharged home. In the period between January 2023 and April 2023, WPH Cares prioritized outreach on a subset of patients expected to have a higher risk of ED returns or a higher likelihood of requiring assistance with navigation of post-hospital discharge. Risk stratification criteria were developed collaboratively by the transitional care team and the ED and were informed by trends in data. This included patients aged <2 or >65, non-English speaking patients, and patients identified at risk for readmission via the LACE Index [23].

Study of interventions

Key process measures of acute care utilization, including 7-day and 30-day ED return rates, are reported, comparing contacted patients versus those who could not be contacted. These timeframes are frequently used in translational care literature, capture both early and medium-term utilization patterns, and align with our institutional metrics [12-17]. Recognizing there may be social determinants of health impacting contact success, we also reviewed detailed demographic characteristics of discharged patients who were contacted and not contacted.

Measures

The primary outcome of this program was the impact of a telehealth outreach with a successful patient contact on 7-day and 30-day ED utilization rates after the initial ED visit.

Secondary outcomes included: 1) a review of patient demographics on the likelihood of successful contact post-hospital discharge, 2) ideal contact timing to optimize patient contact post-ED discharge, including the interval between discharge and attempted contact and the hour and day of the week.

The 7- and 30-day revisit windows were defined by calendar days following each discharge timestamp. Readmissions crossing midnight were attributed to the discharge date/timestamp, consistent with hospital reporting standards.

Revisit outcomes were limited to returns to the WPH ED within 7 and 30 calendar days of each discharge timestamp. Visits to external hospitals or other systems were not captured due to data access limitations.

Each ED discharge event was treated as an independent observation. Patients with multiple discharges could therefore appear more than once in the dataset. If a patient was not contacted following one visit but was successfully contacted after a subsequent visit, the earlier discharge was classified as an unsuccessful contact for that encounter.

An attempted contact was defined as any documented outbound call, voicemail, or patient-initiated callback recorded in the outreach log. When multiple attempts were made for the same discharge, the first successful contact determined the contact status for that encounter.

Successful contact was defined as any instance in which a WPH Cares RN, APP, or SW spoke directly with the patient or a designated caregiver and completed at least one question from the structured post-discharge assessment.

All analyses were restricted to encounters documented from May 2021 onward, following the hospital’s transition to a single EHR platform. As a result, all encounter data, outreach documentation, and outcome variables originated from the same EHR system, eliminating the need for cross-platform linkage or de-duplication. Each discharge event was uniquely identifiable within the system by its encounter ID and discharge timestamp, ensuring direct correspondence between outreach activity and subsequent ED revisit outcomes.

Statistical analysis

To evaluate the impact of the WPH Cares transitional care program on the reduction of ED visit rates and to understand the optimal timing for the post-discharge contact, several statistical methods were employed. The data frame for this study was generated using Python software to identify patients who met the study criteria. A descriptive statistical analysis was conducted by calculating the frequencies and percentages for each demographic characteristic identified within the data set, including primary outcomes for each group. Logistic regression models were designed to evaluate the association between successful WPH Cares contact and the likelihood of an ED return within 7 and 30 days. The primary models included contact status as the main predictor and sex as a covariate, which improved model fit (Akaike’s Information Criterion (AIC) reduction and higher pseudo-R²). Other demographic variables (e.g., age, language, insurance status, and primary care provider) were analyzed descriptively through chi-square and subgroup analyses rather than entered into the regression model, as the goal of this analysis was exploratory rather than predictive.

Chi-squared tests were performed on the differences in contact rates before and after implementation of the targeted outreach strategy (January-April 2023). Chi-squared tests were also used to evaluate the association between contact timing and the primary outcome. An analysis of the association between the timing of the call by time of day and day of week on the likelihood of successful contact was completed through heatmaps.

The statistical analyses for the descriptive statistics, logistic regression analysis, and chi-square tests were performed using a combination of R and Excel, with a statistical significance set at p<0.05. The time of day and day of week heatmap analysis was generated using Python software.

## Results

Study of the WPH Cares program took place for all patients discharged from the ED to home between May 2021 and April 2023 (n=106,888). During this period, WPH Cares attempted to contact 93,933 (87.9%) of those patients. Inability to attempt patient contact was due to patient volume and staffing constraints. Among those discharged home from the ED during this timeframe, 52,210 (48.8%) were successfully contacted.

Table 1 describes the demographics of all patients discharged from the ED in the study timeframe, and the associated attempt rate and contact rate for each demographic factor. Several factors were associated with higher successful contact rates including female sex English as primary language, White race, Asian, Hawaiian, Pacific Islander race, American Indian race, ethnicity listed as “Not Spanish/Hispanic/Latino”, commercial insurance, Medicare insurance, insurance status of “other”, having an identified primary care provider, and older age defined as 60 years or above.

**Table 1:** Characteristics of patients discharged from ED (May 2021 - April 2023) To calculate p-values, either the chi-square or the t-test was used. Chi-square is reported using “X2”, and the t-test is reported using “t”.

	Overall	Contacted	Not Contacted	Attempt Rate	Contact Rate	Statistical Test	P Value
Characteristic	n (%)	n (%)	n(%)	%	% (95 CI)	-	-
Total	106,888 (100%)	52,210 (48.8%)	54,678 (51.2%)	93,933 (87.9%)	-	-	-
Sex	-	-	-	-	-	X^2^= 511.1	<0.0001
Female	59,145 (55.3%)	30,727 (28.7%)	28,418 (26.6%)	52,415 (88.6%)	52.0% (51.6-52.4)	-	-
Male	47,743 (44.7%)	21,483 (20.1%)	26,260 (24.6%)	41,518 (87.0%)	45.0% (44.6-45.6)	-	-
Language	-	-	-	-	-	X^2^= 505.7	<0.0001
English	92,669 (86.7%)	46,472 (43.5%)	46,197 (43.2%)	81,595 (88.1%)	50.1% (49.8-50.4)	-	-
Spanish	12,959 (12.1%)	5,133 (4.8%)	7,826 (7.3%)	11,218 (86.6%)	39.6% (38.8-40.4)	-	-
Other Language	1,260 (1.2%)	605 (0.6%)	655 (0.6%)	1,120 (88.8%)	48.0% (45.2-50.8)	-	-
Race	-	-	-	-	-	X^2^= 482.5	<0.0001
White	48,248 (45.1%)	25,183 (23.6%)	23,065 (21.6%)	42,869 (88.9%)	52.2% (51.8-52.7)	-	-
Black or African American	19,573 (18.3%)	8,827 (8.3%)	10,746 (10.1%)	17,012 (86.9%)	45.1% (44.4-45.8)	-	-
Asian, Hawaiian, or Pacific Islander	3,033 (2.8%)	1,636 (1.5%)	1,397 (1.3%)	2,688 (88.6%)	53.9% (52.1-55.7)	-	-
Native American	185 (0.2%)	100 (0.1%)	85 (0.1%)	172 (93.0%)	54.1% (46.9-61.3)	-	-
Other or Unknown	35,849 (33.5%)	16,464 (15.4%)	19,385 (18.1%)	31,192 (87.0%)	45.9% (45.4-46.4)	-	-
Ethnicity	-	-	-	-	-	X^2^= 345.2	<0.0001
Spanish/Hispanic/Latino	31,679 (29.6%)	14,152 (13.2%)	17,527 (16.4%)	27,475 (86.7%)	44.7% (44.2-45.3)	-	-
Not Spanish/Hispanic/Latino	64,866 (60.7%)	33,089 (31.0%)	31,777 (29.7%)	57,309 (88.3%)	51.0% (50.6-51.4)	-	-
Declined/Unavailable/Unknown	10,343 (9.7%)	4,969 (4.6%)	5,374 (5.0%)	9,149 (88.5%)	48.0% (47.0-49.0)	-	-
Insurance	-	-	-	-	-	X^2^= 1296.3	<0.0001
Commercial	40,699 (38.1%)	20,849 (19.5%)	19,850 (18.6%)	35,890 (88.1%)	51.2% (50.7-51.7)	-	-
Medicare	22,129 (20.7%)	12,400 (11.6%)	9,729 (9.1%)	19,896 (90.0%)	56.0% (55.4-56.7)	-	-
Medicaid	25,652 (24.0%)	11,043 (10.3%)	14,609 (13.7%)	22,073 (86.0%)	43.0% (42.4-43.6)	-	-
Emergency Medicaid	3,558 (3.3%)	1,452 (1.4%)	2,106 (2.0%)	3,012 (84.7%)	40.8% (39.2-42.4)	-	-
Other	3,132 (2.9%)	1,660 (1.6%)	1,472 (1.2%)	2,799 (89.4%)	53.0% (51.3-54.8)	-	-
None	11,718 (11.0%)	4,806 (4.5%)	6,912 (6.5%)	10,263 (87.6%)	41.0% (40.1-41.9)	-	-
Primary Care Provider Listed	-	-	-	-	-	X^2^= 1207.3	<0.0001
Yes	82,899 (77.6%)	42,862 (40.1%)	40,037 (37.5%)	73,351 (88.5%)	51.7% (51.4-52.0)	-	-
No	23,989 (22.4%)	9,348 (8.7%)	14,641 (13.7%)	20,582 (85.8%)	39.0% (38.4-39.6)	-	-
Age Group	-	-	-	-	-	X^2^= 1004.3	<0.0001
0-9	8,915 (8.3%)	3,851 (3.6%)	5,064 (4.7%)	7,646 (85.8%)	43.2% (42.3-44.0)	-	-
10-19	8,549 (8.0%)	3,643 (3.4%)	4,906 (4.6%)	7,241 (84.7%)	42.6% (41.8-43.5)	-	-
20-29	14,617 (13.7%)	6,765 (6.3%)	7,852 (7.3%)	12,891 (88.2%)	46.3% (45.6-47.1)	-	-
30-39	16,119 (15.1%)	7,643 (7.2%)	8,476 (7.9%)	14,058 (87.2%)	47.4% (46.0-47.7)	-	-
40-49	13,827 (12.9%)	6,550 (6.1%)	7,277 (6.8%)	12,121 (87.7%)	47.4% (46.2-47.9)	-	-
50-59	14,604 (13.7%)	6,955 (6.5%)	7,649 (7.2%)	12,722 (87.1%)	47.6% (47.2-48.9)	-	-
60-69	12,250 (11.5%)	6,344 (5.9%)	5,906 (5.5%)	10,903 (89.0%)	51.8% (50.9-52.7)	-	-
70-79	9,115 (8.5%)	5,123 (4.8%)	3,992 (3.7%)	8,259 (90.6%)	56.2% (54.1-57.8)	-	-
80-89	6,397 (6.0%)	3,812 (3.6%)	2,585 (2.4%)	5,845 (91.4%)	59.6% (59.3-62.2)	-	-
90-99	2,388 (2.2%)	1,460 (1.4%)	928 (0.8%)	2,146 (89.9%)	61.1% (58.8-63.1)	-	-
100+	107 (0.1%)	64 (0.1%)	43 (0.04%)	101 (94.4%)	59.8% (34.5-72.4)	-	-
Length of Stay (hours)	-	-	-	-	-	t= -6.56	<0.0001
Mean	3.2	3.3	3.2	-	-	-	-
Median	2.7	2.9	2.6	-	-	-	-

To better understand optimal timing of patient contact, an analysis of the association between post-discharge contact timing (e.g., <24, 24-48, 48-72, 72+ hours, and not contacted) and ED returns within 7 and 30 days was performed (Table 2). Post-discharge contact timing analysis reveals that those who were not contacted by WPH Cares after ED discharge had the highest likelihood of returning to the ED both within 7 and 30 days. Those who were contacted within 24 hours of ED presentation also had a higher likelihood of returning to the ED compared to those contacted between 24 and 48 hours after ED presentation. The group with the lowest return rate was contacted >72 hours after ED presentation.

**Table 2:** Post-discharge contact timing and ED returns To calculate p-values, the chi-square was used. Chi-square is reported using “X^2^”.

	<24 Hours	24-48 Hours	48-72 Hours	>72 Hours	Total Contacted	Not Contacted	ED Return in 7 Days	ED Return in 30 Days	Statistical Test	P Value
	n (%)	n (%)	n (%)	n (%)	n (%)	n (%)	n (%)	n (%)	-	-
Characteristic	14,242	16,289	12,373	9,306	52,210	54,678	5,651	12,217	-	-
Sex	-	-	-	-	-	-	-	-	X^2^= 518.8	<0.0001
Female (n= 59,145)	8,282 (58.2%)	9,624 (59.1%)	7,250 (58.6%)	5,571 (59.9%)	30.727 (58.9%)	28,418 (52.0%)	2,727 (4.6%)	6,121 (10.3%)	-	-
Male (n= 47,743)	5,960 (41.8%)	6,665 (40.9%)	5,123 (41.4%)	3,735 (40.1%)	21,483 (41.1%)	26,260 (48.0%)	2,924 (6.1%)	6,096 (12.8%)	-	-
Language	-	-	-	-	-	-	-	-	X^2^= 522.1	<0.0001
English (n= 92,668)	12,581 (88.3%)	14,619 (89.7%)	10,985 (88.8%)	8,287 (89.1%)	46,472 (89.0%)	46,196 (84.4%)	4,965 (5.4%)	10,694 (11.5%)	-	-
Spanish (n=12,959)	1,498 (10.5%)	1,489 (9.1%)	1,247 (10.1%)	899 (9.7%)	5,133 (9.8%)	7,826 (14.3%)	653 (5.0%)	1,429 (11.0%)	-	-
Other Language (n=1,261)	163 (1.1%)	181 (1.1%)	141 (1.1%)	120 (1.3%)	605 (1.2%)	656 (1.2%)	33 (2.6%)	94 (7.5%)	-	-
Race	-	-	-	-	-	-	-	-	X^2^= 522.4	<0.0001
White (n= 48,248)	6,692 (47.0%)	7,975 (49.0%)	6,051 (48.9%)	4,465 (48.0%)	25,183 (48.2%)	23,065 (42.2%)	2,478 (5.1%)	5,084 (10.5%)	-	-
Black or African American (n= 19,573)	2,353 (16.5%)	2,826 (17.3%)	2,110 (17.1%)	1,538 (16.5%)	8,827 (16.9%)	10,746 (19.7%)	1,358 (6.9%)	3,217 (16.4%)	-	-
Asian, Hawaiian, or Pacific Islander (n= 3,033)	449 (3.2%)	475 (2.9%)	379 (3.1%)	333 (3.6%)	1,636 (3.1%)	1,397 (2.6%)	130 (4.3%)	245 (8.1%)	-	-
Native American (n= 185)	28 (0.2%)	31 (0.2%)	20 (0.2%)	21 (0.2%)	100 (0.2%)	85 (0.2%)	8 (4.3%)	15 (8.1%)	-	-
Other or Unknown (n= 35,849)	4,720 (33.1%)	4,982 (30.6%)	3,813 (30.8%)	2,949 (31.7%)	16,464 (31.5%)	19,385 (35.5%)	1,677 (4.7%)	3,656 (10.2%)	-	-
Ethnicity	-	-	-	-	-	-	-	-	X^2^= 422.8	<0.0001
Spanish/Hispanic/Latino (n=31,679)	3,930 (27.6%)	4,285 (26.3%)	3,365 (27.2%)	2,572 (27.6%)	14,152 (27.1%)	17,527 (32.1%)	1,761 (5.6%)	3,786 (12.0%)	-	-
Not Spanish/Hispanic/Latino (n= 64,866)	8,728 (61.3%)	10,494 (64.4%)	7,961 (64.3%)	5,906 (63.5%)	33,089 (63.4%)	31,777 (58.1%)	3,636 (5.6%)	7,867 (12.1%)	-	-
Declined/Unavailable/Unknown (n= 10,343)	1,584 (11.1%)	1,510 (9.3%)	1,047 (8.5%)	828 (8.9%)	4,969 (9.5%)	5,374 (9.8%)	254 (2.5%)	564 (5.5%)	-	-
Insurance	-	-	-	-	-	-	-	-	X^2^= 1615.0	<0.0001
Commercial (n= 40,699)	5,743 (40.3%)	6,359 (39.0%)	4,938 (39.9%)	3,809 (40.9%)	20,849 (39.9%)	19,850 (36.3%)	1,524 (3.7%)	3,249 (8.0%)	-	-
Medicare (n= 22,129)	3,501 (24.6%)	3,980 (24.4%)	2,915 (23.6%)	2,004 (21.5%)	12,400 (23.8%)	6,912 (17.8%)	1,336 (6.0%)	3,056 (13.8%)	-	-
Medicaid (n= 25,652)	3,278 (23.0%)	3,363 (20.6%)	2,566 (20.7%)	1,836 (19.7%)	11,043 (21.2%)	14,609 (26.7%)	1,975 (8.7%)	4,248 (16.6%)	-	-
Emergency Medicaid (n= 3,558)	420 (2.9%)	442 (2.7%)	324 (2.6%)	266 (2.9%)	1,452 (2.8%)	2,106 (3.9%)	277 (7.8%)	581 (16.3%)	-	-
Other (n= 3,132)	482 (3.4%)	532 (3.3%)	378 (3.1%)	268 (2.9%)	1,660 (3.2%)	1,472 (2.7%)	129 (4.1%)	255 (8.1%)	-	-
None (n= 11,718)	818 (5.7%)	1,613 (9.9%)	1,252 (10.1%)	1,123 (12.1%)	4,806 (9.2%)	6,912 (12.6%)	410 (3.5%)	828 (7.1%)	-	-
Primary Care Provider Listed	-	-	-	-	-	-	-	-	X^2^= 1247.5	<0.0001
Yes (n =82,899)	11,470 (80.5%)	13,330 (81.8%)	10,285 (83.1%)	7,777 (83.6%)	42,862 (82.0%)	40,037 (73.2%)	4,531 (5.5%)	9,967 (12.0%)	-	-
No (n= 23,989)	2,772 (19.5%)	2,959 (18.2%)	2,088 (16.9%)	1,529 (16.4%)	9,348 (17.9%)	14,641 (26.8%)	1,120 (4.7%)	2,250 (9.4%)	-	-
Age Group	-	-	-	-	-	-	-	-	X^2^= 1,286.5	<0.0001
0-9 (n= 8,915)	1,080 (7.6%)	1,096 (6.7%)	881 (7.1%)	794 (8.5%)	3,851 (7.4%)	5,064 (9.3%)	269 (3.0%)	656 (7.4%)	-	-
10-19 (n= 8,549)	1,095 (7.7%)	1,076 (6.6%)	819 (6.6%)	653 (7.0%)	3,643 (7.0%)	4,906 (9.0%)	299 (3.5%)	598 (7.0%)	-	-
20-29 (n= 14,617)	2,130 (15.0%)	2,151 (13.2%)	1,432 (11.6%)	1,052 (11.3%)	6,765 (13.0%)	7,852 (14.4%)	817 (5.6%)	1,757 (12.0%)	-	-
30-39 (n= 16,119)	2,059 (14.5%)	2,463 (15.1%)	1,774 (14.3%)	1,347 (14.5%)	7,643 (14.6%)	8,476 (15.5%)	946 (5.9%)	2,090 (13.0%)	-	-
40-49 (n= 13,827)	1,686 (11.8%)	2,034 (12.5%)	1,616 (13.1%)	1,214 (13.0%)	6,550 (12.5%)	7,277 (13.3%)	868 (6.3%)	1,740 (12.6%)	-	-
50-59 (n= 14,604)	1,711 (12.0%)	2,113 (13.0%)	1,791 (14.5%)	1,340 (14.4%)	6,955 (13.3%)	7,649 (14.0%)	970 (6.6%)	1,971 (13.5%)	-	-
60-69 (n=12,250)	1,553 (10.9%)	1,955 (12.0%)	1,625 (13.1%)	1,211 (13.0%)	6,344 (12.2%)	5,906 (10.8%)	633 (5.2%)	1,432 (11.7%)	-	-
70-79 (n= 9,115)	1,333 (9.4%)	1,635 (10.0%)	1,265 (10.2%)	890 (9.6%)	5,123 (9.8%)	3,992 (7.3%)	432 (4.7%)	976 (10.7%)	-	-
80-89 (n= 6,397)	1,117 (7.8%)	1,250 (7.7%)	869 (7.0%)	576 (6.2%)	3,812 (7.3%)	2,585 (4.7%)	309 (4.8%)	707 (11.1%)	-	-
90-99 (n= 2,388)	457 (3.2%)	493 (3.0%)	289 (2.3%)	221 (2.4%)	1,460 (2.8%)	928 (1.7%)	105 (4.4%)	282 (11.8%)	-	-
100+ (n= 107)	21 (0.1%)	23 (0.1%)	12 (0.1%)	8 (0.1%)	64 (0.1%)	43 (0.1%)	3 (2.8%)	8 (7.5%)	-	-
ED Return in 7 Days (n =5,651)	804 (5.6%)	731 (4.5%)	433 (3.5%)	196 (2.1%)	2,164 (4.1%)	3,487 (6.4%)	-	-	X^2^= 421.2	<0.0001
ED Return in 30 Days (n= 12,217)	1,666 (11.7%)	1,684 (10.3%)	1,173 (9.5%)	718 (7.7%)	5,241 (10.0%)	6,976 (12.8%)	-	-	X^2^= 288.9	<0.0001

Among the entire discharged population, regardless of post-discharge contact timing, factors associated with a higher likelihood of ED return visits within 7 days were Male sex, English as preferred language, African American race, Medicare insurance, Medicaid insurance, Emergency Medicaid insurance, having a primary care provider, and 20-59 years of age. Factors associated with a higher likelihood of ED return visits within 30 days were similar, with minor differences in associated age brackets.

Patients successfully contacted by the WPH Cares team were less likely to return to the ED within 7 days compared to those not contacted (OR 0.69, p<0.0001). Similarly, patients successfully contacted by the WPH Cares team were less likely to return to the ED within 30 days compared to those not contacted OR 0.82, p<0.0001). Of note, because ED revisit outcomes were relatively common (10-13%), odds ratios may overstate the absolute effect size compared with risk ratios.

Subgroup analysis provided in Table 3 demonstrates the initial WPH Cares strategy of attempting to contact all patients discharged from the ED, which ended in December 2022. From January 2023 to April 2023, a targeted strategy was implemented to prioritize outreach for a subset of patients expected to have a higher risk of ED returns, as described in “Key Changes in Workflow from May 2021 - April 2023” in Table 4.

**Table 3:** Subgroup analysis from May 2021 to December 2022, when WPH Cares was able to attempt to contact all patients discharged from the ED To calculate p-values, either chi-square or ANOVA tests were used. Chi-square is reported using “X^2^”, and ANOVA testing is reported using “F”. WPH: White Plains Hospital

May 2021- December 2022	Overall	Contacted	Not Contacted	Attempt Rate	Contact Rate	ED Return in 7 Days	ED Return in 30 Days	Statistical Test	P Value
Characteristic	n (%)	n (%)	n(%)	n(%)	% (95 CI)	n(%)	-	-	-
Total	88,132 (100%)	42,190 (47.9%)	45,942 (52.1%)	80,376 (91.2%)	-	4,733 (5.4%)	10,356 (11.8%)	-	-
Sex	-	-	-	-	-	-	-	X^2^= 325.5	<0.0001
Female	48,521 (55.1%)	24,559 (27.9%)	23,962 (27.2%)	44,457 (91.6%)	50.6% (50.2-51.0)	2,278 (4.7%)	5,138 (10.6%)	-	-
Male	39,611 (44.9%)	17,631 (20.0%)	21,980 (24.9%)	35,919 (90.7%)	44.5% (44.0-41.5)	2,455 (6.2%)	5,218 (13.2%)	-	-
Language	-	-	-	-	-	-	-	X^2^= 251.4	<0.0001
English	76,539 (86.8%)	37,405 (42.4%)	39,134 (44.4%)	69,818 (91.2%)	48.9% (48.6-49.3)	4,180 (5.5%)	9,079 (11.9%)	-	-
Spanish	10,534 (12.0%)	4,276 (4.9%)	6,258 (7.1%)	9,579 (90.1%)	40.6% (39.7-41.5)	524 (5.0%)	1,196 (11.4%)	-	-
Other Language	1,059 (1.2%)	509 (0.6%)	550 (0.6%)	979 (92.4%)	48.1% (45.1-51.1)	29 (2.7%)	81 (7.6%)	-	-
Race	-	-	-	-	-	-	-	X^2^=299.7	<0.0001
White	40,046 (45.4%)	20,264 (23.0%)	19,782 (22.4%)	36,680 (91.6%)	50.6% (50.1-51.1)	2,086 (5.2%)	4,288 (10.7%)	-	-
Black or African American	16,174 (18.4%)	7,105 (8.1%)	9,069 (10.3%)	14,611 (90.3%)	43.4% (42.6-44.2)	1,135 (7.0%)	2,755 (17.0%)	-	-
Asian, Hawaiian, or Pacific Islander	2,442 (2.8%)	1,306 (1.5%)	1,136 (1.3%)	2,245 (91.2%)	53.5% (51.5-55.5)	115 (4.7%)	210 (8.6%)	-	-
Native American	161 (0.2%)	82 (0.1%)	79 (0.1%)	153 (95.0%)	50.9% (43.2-58.6)	8 (5.0%)	15 (9.3%)	-	-
Other or Unknown	29,309 (33.3%)	13,433 (15.2%)	15,876 (18.0%)	26,687(91.1%)	45.8% (45.2-46.4)	1,389 (4.7%)	3,088 (10.5%)	-	-
Ethnicity	-	-	-	-	-	-	-	X^2^= 166.1	<0.0001
Spanish/Hispanic/Latino	25,641 (29.1%)	11,442 (13.0%)	14,199 (16.1%)	23,337 (91.0%)	44.6% (44.0-45.2)	1,456 (5.7%)	3,178 (12.4%)	-	-
Not Spanish/Hispanic/Latino	53,549 (60.8%)	26,521 (30.1%)	27,028 (30.7%)	48,884 (91.3%)	49.0% (48.6-49.2)	3,046 (5.7%)	6,664 (12.4%)	-	-
Declined/Unavailable/Unknown	8,942 (10.1%)	4,227 (4.8%)	4,715 (5.3%)	8,155 (91.2%)	47.3% (46.3-48.3)	231 (2.6%)	514 (5.7%)	-	-
Insurance	-	-	-	-	-	-	-	X^2^=860.5	<0.0001
Commercial	33,416 (37.9%)	16,721 (19.0%)	16,695 (18.9%)	30,621 (91.6%)	50.0% (49.5-50.5)	1,290 (3.9%)	2,745 (8.2%)	-	-
Medicare	18,241 (20.7%)	9,892 (11.2%)	8,349 (9.5%)	16,721 (91.7%)	54.2% (53.5-54.9)	1,121 (6.1%)	2,585 (14.2%)	-	-
Medicaid	21,107 (23.9%)	9,018 (10.2%)	12,089 (13.7%)	19,037 (90.1%)	42.7% (42.0-43.4)	1,628 (7.7%)	3,600 (17.1%)	-	-
Emergency Medicaid	2,814 (3.2%)	1,185 (1.3%)	1,629 (1.8%)	2,525 (89.7%)	42.1% (40.3-43.9)	224 (8.0%)	494 (17.6%)	-	-
Other	2,554 (2.9%)	1,335 (1.5%)	1,219 (1.4%)	2,382 (93.3%)	52.3% (50.4-54.2)	104 (4.1%)	210 (8.2%)	-	-
None	10,000 (11.3%)	4,039 (4.6%)	5,961 (6.8%)	9,090 (90.9%)	40.4% (39.4-41.4)	366 (3.7%)	722 (7.2%)	-	-
Primary Care Provider Listed	-	-	-	-	-	-	-	X^2^= 759.5	<0.0001
Yes	67,893 (77.0%)	34,226 (38.8%)	33,667 (38.2%)	62,159 (91.6%)	50.4% (50.0-50.8)	3,797 (5.6%)	8,438 (12.4%)	-	-
No	20,239 (23.0%)	7,964 (9.0%)	12,275 (13.9%)	18,217 (90.0%)	39.3% (38.6-40.0)	936 (4.6%)	1,918 (9.5%)	-	-
Age	-	-	-	-	-	-	-	X^2^= 620.3	<0.0001
0-9	7,267 (8.2%)	3,317 (3.8%)	3,950 (4.5%)	6,665 (91.7%)	45.6% (44.5-46.8)	220 (3.0%)	552 (7.6%)	-	-
10-19	7,056 (8.0%)	3,128 (3.5%)	3,928 (4.5%)	6,479 (91.8%)	44.3% (43.1-45.5)	248 (3.5%)	500 (7.1%)	-	-
20-29	12,233 (13.9%)	5,479 (6.2%)	6,754 (7.7%)	11,122 (90.9%)	44.8% (43.9-45.7)	700 (5.7%)	1,516 (12.4%)	-	-
30-39	13,360 (15.2%)	6,111 (6.9%)	7,249 (8.2%)	12,018 (90.0%)	45.7% (44.9-46.5)	810 (6.1%)	1,792 (13.4%)	-	-
40-49	11,354 (12.9%)	5,188 (5.9%)	6,166 (7.0%)	10,276 (90.5%)	45.7% (44.8-46.6)	742 (6.5%)	1,488 (13.1%)	-	-
50-59	12,051 (13.7%)	5,574 (6.3%)	6,477 (7.3%)	10,907 (90.5%)	46.3% (45.4-47.2)	806 (6.7%)	1,677 (13.9%)	-	-
60-69	10,081 (11.4%)	5,112 (5.8%)	4,969 (5.6%)	9,296 (92.2%)	50.7% (49.7-51.7)	511 (5.1%)	1,181 (11.7%)	-	-
70-79	7,426 (8.4%)	4,026 (4.6%)	3,400 (3.9%)	6,909 (93.0%)	54.2% (53.1-55.3)	363 (4.9%)	833 (11.2%)	-	-
80-89	5,239 (5.9%)	3,031 (3.4%)	2,208 (2.5%)	4,836 (92.3%)	57.9% (56.6-59.2)	245 (4.7%)	574 (11.0%)	-	-
90-99	1,980 (2.2%)	1,177 (1.3%)	803 (0.9%)	1,788 (90.3%)	59.4% (57.2-61.6)	86 (4.3%)	237 (12.0%)	-	-
100+	85 (0.1%)	47 (0.1%)	38 (0.04%)	80 (94.1%)	55.3% (44.7-65.9)	2 (2.4%)	6 (7.1%)	-	-
Length of Stay (hours)	-	-	-	-	-	-	-	F= 35.0	<0.0001
Mean	3.2(-)	3.2(-)	3.1(-)	-	-	-	-	-	-
Median	2.7(-)	2.8(-)	2.6(-)	-	-	-	-	-	-

The targeted outreach strategy resulted in the overall attempt rate falling from 91.2% to 72.3% (p<0.0001) with an increase in overall contact rate from 47.9% to 53.4% (p<0.0001).

In the targeted strategy cohort, younger patients (0-19 years old) had a lower attempt (91.8% vs 55.5%, p<0.0001) and contact rate (45.0% vs 33.4%, p<0.0001). The elderly population (age 70 and older) also had a lower attempt rate (92.4% to 83.6%, p<0.0001) but higher contact rate (56.2% to 66.5%, p<0.0001) in the targeted outreach cohort. The attempt and contact rates for non-English speaking patients similarly fell.

As discussed in "Key Changes in Workflow from May 2021 - April 2023," during the targeted outreach period, the WPH Cares team implemented operational refinements designed to cope with increasing patient volume and to prioritize high-risk groups. In addition, call timing was strategically shifted toward late afternoon and early evening hours when patient responsiveness was highest, and daily call lists were streamlined to focus on these targeted groups. The combination of data-driven patient selection and optimized call timing may explain the increased proportion of successful contacts despite fewer total call attempts.

When comparing the ED return rates between the two outreach groups, the 7-day return rate was lower in the targeted strategy group (5.4% to 4.9%, p<0.0001). The results were similar for the 30-day returns between these two groups (11.8% to 9.9%, p<0.0001). This may be due to the workflow changes detailed above. The workflow changes may have resulted in the observed increased contact rates in the targeted strategy group among older patients, Black/African American patients, those with Medicare, Emergency Medicaid, and no insurance.

**Figure 1:**
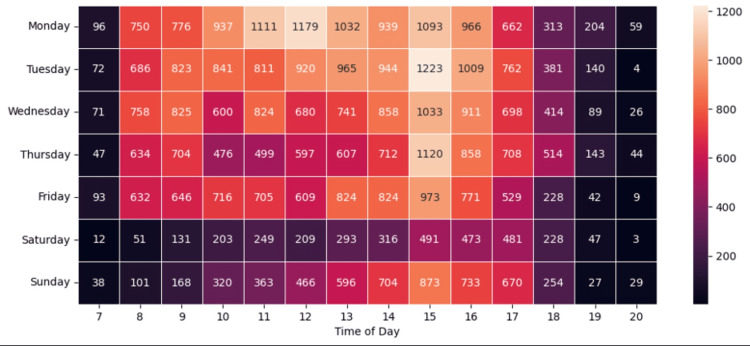
WPH cares post-discharge contacts by time of day and day of week WPH: White Plains Hospital

**Figure 2:**
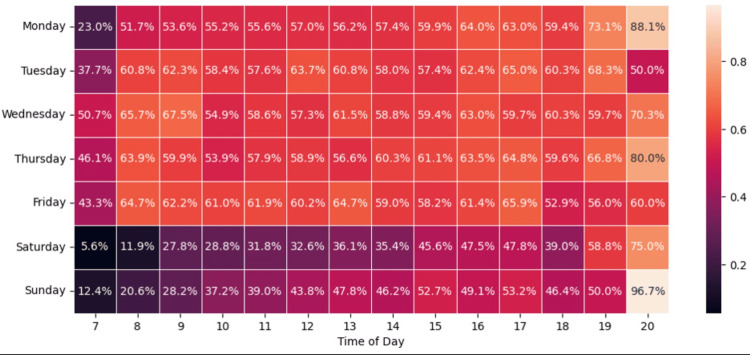
WPH Cares post-discharge contact success rate by time of day and day of week WPH: White Plains Hospital

Lastly, we looked at the times of day and week associated with the highest successful contact rates. Figures 1-2 show heatmaps of contact rates and contact success rates by time of day and day of week. Patients were more likely to be successfully contacted (e.g., they picked up the phone) on weekdays compared to weekends (59.6% vs 41.1%, p<0.0001) and more likely to be successfully contacted in the late afternoon or evening compared to the morning and early afternoon (57.5% vs 53.7%, p<0.0001).

## Discussion

Patients who were discharged from the ED and were successfully contacted by the WPH Cares transitional care program had a lower chance of return ED visit within 7 and 30 days. When the ED volume surpassed the comprehensive outreach capabilities of the WPH Cares team, this had an impact on return ED visits held for the targeted outreach group, which was a higher-risk group. This finding lends support to the theory that ED transitional care programs that provide services beyond a simple RN callback and offer a robust set of services can avoid preventable ED return visits [18]. We suspect that the reduction of ED revisits among patients contacted by WPH Cares is due to the variety of services that it offers, including the ability to answer clinical questions regarding home care and prescriptions, assistance in scheduling timely follow-up appointments, referrals to telehealth, mental health and palliative care, paramedicine visits, IV antibiotic or fluid administration, care management for medically complex patients, home and transportation services, and charity care. This supports the existing literature that post-ED transitional care programs that can connect patients to a clinician or a robust care pathway can reduce ED visits [16,17].

While other studies have focused on outcomes of ED patients enrolled in post-discharge transition of care programs, no previous research to our knowledge has been published on the ideal time to contact discharged ED patients [12-17]. In our study, progressively longer post-discharge contact times had progressively lower ED return rates. The ideal time to contact discharged ED patients appears to be >72 hours after discharge, with that cohort showing the lowest rate of ED returns within 7 and 30 days. However, caution is needed in interpreting causality due to demographic differences and potential unmeasured confounders. Several explanations exist for the ideal timing result. Included in the group contacted <24 hours after ED discharge were those who were referred for close follow-up by the ED or even those who called WPH Cares themselves (WPH Cares contact information is clearly indicated on ED discharge paperwork). Therefore, the group that was contacted in <24 hours may be medically needier and thus have higher ED revisit rates. Another potential explanation is that after ED discharge, there may be a “sweet spot” where enough uncertainty exists that a post-discharge outreach can steer patients away from an ED revisit. If patients are contacted too early, there may not have been adequate time for questions regarding post-discharge care or for worsening symptoms to emerge, resulting in a repeat ED visit. However, if patients are contacted too late, the opportunity to address their concerns may have passed, which could result in an ED visit. Further research could study whether a subset of patients would benefit from an early post-discharge contact, and what exact time in the >72-hour post-discharge period is ideal to contact patients.

There are several limitations to this analysis, including data limitations such as the inability to access revisit data from other institutions, confounding, generalizability, and the COVID-19 pandemic, which may have caused variation over time in ED utilization and outreach success. Future analyses will incorporate a fully adjusted multivariable model or propensity-matched design to better control for many of these factors.

Successfully contacted patients differ demographically from patients that WPH Cares was unable to contact. Patients who were more likely to be contacted were more likely to be female, speak English as their primary language, identify as White, Asian, Hawaiian, Pacific Islander, or American Indian, “Not Spanish/Hispanic/Latino,” have commercial insurance or Medicare, have a primary care provider, and be over the age of 60. Other potential confounders that were not measured may include, but are not limited to, socioeconomic status, health literacy, employment status, and whether they are caregivers for others.

A study that looked at risk factors for admission within 72 hours of ED discharge noted that elderly patients and patients with Medicare insurance were at particularly high risk [24]. This suggests that WPH Cares was successful in contacting elderly patients who were at risk of ED revisit and admission, and could account for some of the reduced ED revisit rate noted among the contacted group.

However, the demographic differences and potential confounders listed above call into question the argument that contact by WPH Cares was the causal factor in reduced ED returns, and reflect the possibility that the group that was successfully contacted had lower ED revisits because they have more pre-existing socioeconomic privilege, access to care outside of the ED, language skills or health literacy that may prevent revisiting the hospital. While the WPH Cares program targets populations at risk for ED revisit and routinely utilizes certified medical interpreters for all non-English-speaking patients, this analysis highlighted the need to address several remaining gaps in outreach.

To further remove systemic barriers to contacting patients, we have implemented additional logic-based prioritization for ED callbacks that incorporates ED length of stay and allows providers to flag higher-acuity patients for expedited outreach. We also introduced a process for sending a secure message via the electronic medical record (EMR) to patients who have not been reached within three days of discharge, providing them with the opportunity to contact WPH Cares directly for assistance. These interventions collectively aim to enhance equitable access, timeliness, and engagement across patient populations, ensuring that language, acuity, and technology barriers are systematically addressed in future iterations of the program.

Finally, due to changing EMRs and multiple unavoidable confounding variables, including the COVID-19 pandemic and increasing ED and hospital volume, we were unable to compare ED return rates from before and after the implementation of the WPH Cares program to determine if the program resulted in decreased ED returns. The non-randomized nature of this study design further limits the conclusions that can be drawn. A future randomized, controlled study, including pre-implementation data collection, would be helpful to account for these limitations.

## Conclusions

The WPH Cares telehealth-based transitional care program was associated with reduced repeat ED visits at 7 and 30 days after the index ED visit, but causal relationships cannot be inferred due to potential confounding and selection bias. Socially vulnerable populations-particularly non-English - speaking and uninsured patients - were less likely to be reached, underscoring the need for more proactive, equity-focused outreach strategies in future program iterations.

We also demonstrated that the highest success rate in contacting patients after ED discharge appears to be on a weekday, in the late afternoon or evening.

Future research could incorporate propensity-based modeling to better control for confounding and evaluate causal relationships. Further research could also evaluate the economic outcomes of the WPH Cares model.
